# Editorial: Measuring vaccination perception, confidence, and uptake: advancing instrument development and validation

**DOI:** 10.3389/fpubh.2026.1993684

**Published:** 2026-10-07

**Authors:** Divya S. Subramaniam, Aida Bianco, Dipti P. Subramaniam

**Affiliations:** 1 Trudy Busch Valentine School of Nursing, Saint Louis University, Saint Louis, MO, United States; 2 Duo Scientia, LLC, St. Louis, MO, United States; 3 Department of Medical and Surgical Science, University “Magna Græcia” of Catanzaro, Catanzaro, Italy; 4 IntegrityCE, LLC, Winter Garden, FL, United States

**Keywords:** instrument development, psychometrics, public health, vaccination, vaccine confidence, vaccine hesitancy

Vaccination remains one of the most effective public health interventions available, yet global immunization coverage continues to fall short of the targets set by international health authorities. The World Health Organization has named vaccine hesitancy one of the top 10 threats to global health (1), and vaccine decision-making is commonly framed using the WHO Strategic Advisory Group of Experts' three-component (3C) model of confidence, complacency, and convenience (2). Progress on this front depends on psychometrically sound instruments capable of capturing these constructs validly across populations. This Research Topic, “*Measuring vaccination perception, confidence, and uptake: advancing instrument development and validation*,” was convened to support the development of new instruments, the validation and cross-cultural adaptation of existing tools, and the application of these measures to inform vaccination programs. The 13 articles gathered here (Table 1) cover instrument development, determinants of hesitancy and uptake among healthcare workers and general populations, a regional narrative review, and post-vaccination safety monitoring.

**Table 1:** Characteristics of the 13 contributions to this Research Topic.

References	Country/region	Population	Vaccine(s)	Study design
Subramaniam et al.	United States	General adults	General (multi-vaccine)	Instrument development (content and face validity)
Meiklejohn et al.	United States	General adults	COVID-19	Instrument development (EFA, psychometric evaluation)
Wang et al.	China	Community-dwelling older adults	Influenza	Cross-sectional survey
Zhang P. et al.	China	Healthcare workers	Seasonal influenza	Cross-sectional survey
Lien et al.	Singapore	Healthcare workers	COVID-19	Cross-sectional survey
Aksoy et al.	Türkiye	Community pharmacists	General vaccination	Cross-sectional survey
Deng et al.	China	Middle-aged and older adults	Herpes zoster	Cross-sectional survey (structural equation model)
Heinze et al.	United Kingdom	Healthcare workers	Measles	Qualitative interview study
Zhang M. et al.	China	General residents (nationwide)	General vaccination	Cross-sectional survey
Bui et al.	Vietnam	General public	COVID-19	Cross-sectional survey
Ali et al.	GCC region	Healthcare workers	Influenza	Narrative review
Xu et al.	China	Animal-bite clinic patients	Rabies	Retrospective multivariate analysis
Wu et al.	China	Young adults (18–26 years)	HPV	Cross-sectional survey (PLS-SEM)

## Instrument development and psychometric validation

Two contributions in this Research Topic directly advance the measurement science that motivated this Topic. Subramaniam et al. ground the development of a new general measure of adult vaccine hesitancy for U.S. adults in the 3C framework, reporting content and face validity findings, including a content validity index derived from expert panel review, for an instrument intended to capture hesitancy across vaccine types rather than a single indication. Meiklejohn et al. report the development and psychometric evaluation of the Vaccine Hesitancy and Perception Scale toward COVID-19 vaccination (VHAPS-CV19), employing principal axis factoring with varimax rotation to derive a two-factor structure that distinguishes a hesitancy factor (confidence, complacency, and convenience) from a perception-and-trust factor (perceived benefit, barrier, susceptibility, severity, and trust), together with internal consistency estimates for each subscale. Together, the two studies trace the stages that valid measurement of a construct this complex demands, from item generation and content validity through factor structure and reliability testing, and they leave the field with two complementary tools, one general-purpose and one vaccine-specific, for future validation and cross-cultural adaptation work.

## Hesitancy and uptake among healthcare workers

Several contributions in this Topic examine hesitancy and uptake among healthcare workers, a population whose own vaccination behavior and patient communication directly shape public confidence. Zhang P. et al. investigated uptake, willingness, and practices toward seasonal influenza vaccination among healthcare workers in China using a cross-sectional design. Lien et al., similarly employed a cross-sectional survey to explore attitudes toward COVID-19 vaccination among healthcare workers at a national infectious disease center in Singapore, while Aksoy et al. surveyed community pharmacists in Türkiye regarding their knowledge, attitudes, and barriers toward vaccination, extending the focus to frontline advocates outside the hospital setting. Heinze et al. took a distinct, qualitative approach, interviewing healthcare workers at a London hospital to identify barriers and facilitators to measles vaccination uptake; this design complements the quantitative work elsewhere in the Topic, surfacing workplace, informational, and interpersonal factors that structured surveys tend to miss.

## Hesitancy and uptake in community and general populations

A second cluster of contributions addresses hesitancy and uptake determinants in community and general-population samples, extending the scope of this Topic beyond influenza and COVID-19 to additional vaccine types and life stages. Wang et al. examined influenza vaccine hesitancy and its influencing factors among community-dwelling older adults in Shihezi City, Xinjiang, in western China, a population and region underrepresented in the broader hesitancy literature. Deng et al. studied service accessibility, social norms, and herpes zoster vaccination intention among middle-aged and older adults in Chongqing, applying a mediation framework centered on perceived usefulness. Wu et al. examined factors influencing Chinese young adults' intention to receive HPV vaccination by integrating the Health Belief Model with the Theory of Planned Behavior within a partial least squares structural equation model; in a sample of 516 respondents aged 18 to 26, perceived severity and perceived behavioral control emerged as the strongest direct predictors of vaccination intention, and attitude was confirmed as a significant mediator linking health-belief constructs to intention. At a broader population level, Zhang M. et al. analyzed influencing factors and enhancement strategies for vaccination behavior among Chinese residents using a nationwide survey, and Bui et al. drew lessons from Vietnam's first COVID-19 vaccine rollout regarding hesitancy and misinformation, with direct relevance to future pandemic preparedness.

## Review and vaccine safety considerations

Rounding out the Research Topic, Ali et al. provide a narrative review of enablers and barriers to influenza vaccine uptake among healthcare workers in Gulf Cooperation Council countries, synthesizing evidence from a regional context that is comparatively underrepresented in the vaccine hesitancy literature relative to North America, Europe, and East Asia. Xu et al. contribute a distinct but complementary perspective, presenting a multivariate analysis of risk factors for adverse events following rabies vaccination using real-world data. This work centers on vaccine safety rather than perception, but it is a reminder that confidence in vaccination rests on lived experience with tolerability as much as on attitudes and access, and that perception-focused instruments are best read alongside pharmacovigilance data.

## Methodological and geographic patterns across the Topic

A few patterns stand out across this Research Topic (Figure 1; Table 1). Geographically, contributions concentrate in China (six studies), with additional representation from the United States, Singapore, Türkiye, Vietnam, the United Kingdom, and the Gulf Cooperation Council region; populations in Sub-Saharan Africa, South Asia, and Latin America are absent, which limits how far these findings generalize and points to a direction for future Research Topics. Methodologically, most contributions rely on cross-sectional, self-report surveys. These designs are useful for generating hypotheses about correlates of hesitancy, but they cannot establish temporal precedence between attitude and behavior, and they remain vulnerable to social desirability and recall bias. Only one contribution employed qualitative interview methods, and none employed a longitudinal or experimental design; this concentration of designs mirrors the broader hesitancy literature and marks a gap that future work should close.

**Figure 1:**
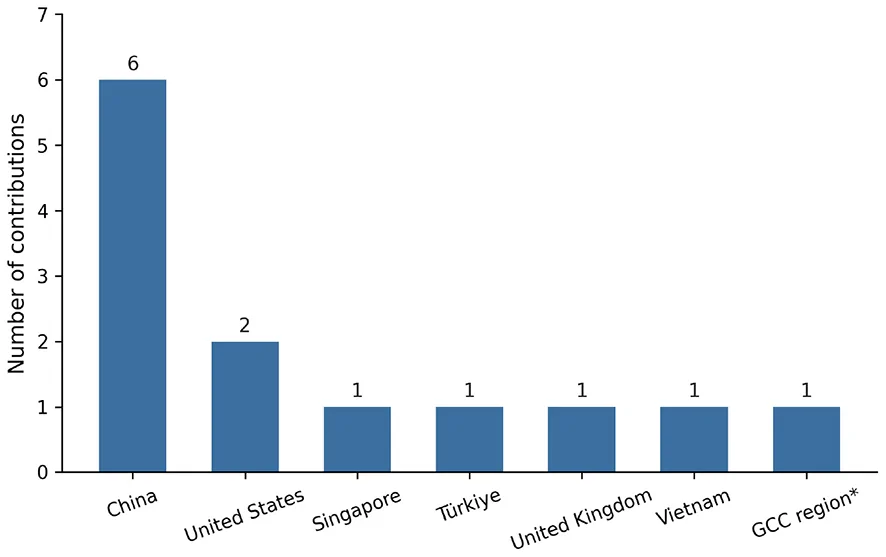
Geographic distribution of the 13 contributions to this Research Topic, by country or region of the study population. *GCC region refers to the Gulf Cooperation Council countries covered by the narrative review of Ali et al.

## Synthesis and future directions

Taken together, the contributions to this Research Topic make clear that vaccine decision-making has many layers. Hesitancy and confidence are shaped at once by individual psychology, professional role, cultural context, service accessibility, and direct experience with vaccine safety. The instrument development studies respond to a real methodological gap: the field needs measurement tools that are psychometrically sound and adaptable across populations. The determinant studies argue the opposite case for context. Findings from healthcare workers in the Gulf region, older adults in western China, and pharmacists in Türkiye are not interchangeable; each offers insight for the setting studied rather than a universal account of vaccine hesitancy.

A few gaps remain for future research. Cross-cultural validation of the instruments introduced here, including measurement invariance testing and comparison against benchmarks such as the WHO SAGE Vaccine Hesitancy Scale (2), would strengthen confidence in their applicability across populations. Longitudinal designs tracking how measured hesitancy or confidence relates to actual vaccination behavior over time, rather than to intention alone, would help close the well-documented gap between attitudinal measurement and uptake outcomes. Greater use of qualitative and mixed-methods designs, alongside the cross-sectional surveys predominant here, would help clarify mechanisms that quantitative models identify but cannot fully explain. Finally, as vaccine platforms and communication environments evolve, measurement tools will need periodic re-validation to remain sensitive to emerging determinants of hesitancy.

We thank all contributing authors for their rigorous work and all reviewers for their careful evaluation of these manuscripts. We hope this Research Topic serves as a resource for researchers and practitioners seeking to measure, understand, and ultimately improve vaccine confidence and uptake worldwide.
